# Utilization of multifrequency permittivity measurements in addition to biomass monitoring

**DOI:** 10.1186/1753-6561-5-S8-O10

**Published:** 2011-11-22

**Authors:** Christoph Heinrich, Tim Beckmann, Heino Büntemeyer, Thomas Noll

**Affiliations:** 1 Institute of Cell Culture Technology, Bielefeld University, 33615 Bielefeld, Germany

## Introduction

In recent years measurement of permittivity signal has been increasingly used for online biomass monitoring of cell cultures. Breweries use it as an established method for fermenter inoculation and bioprocess control for instance [1]. In the case of animal cell cultures the correlation between permittivity and viable cell densities determined offline varies along cultivation time. Hence, several authors have used the permittivity signal as an indirect method for measuring oxygen and glutamine consumption as well as intracellular nucleotide phosphate concentrations [2,3]. The latest generation of biomass monitoring devices allows parallel measurement of permittivity at a range of frequencies leading to an improvement in the correlation between biomass and permittivity and providing a tool to further explore other aspects related to the physiological state of the cells.

## Material and methods

In this study 10 different cell lines, among them industrial cell lines as well as cell lines distributed by ATCC, were cultivated. Cell type specific chemically defined and animal component-free media (TeutoCell AG) were used. Batch, fed-batch and perfusion bioreactor cultivations were carried out in controlled benchtop vessels (Sartorius AG or Applikon Biotechnology). The i-Biomass 465 sensor (FOGALE nanotech) was used for online multifrequency permittivity monitoring. In addition the permittivity signal was used to implement a fully automated cell bleed to maintain a constant viable cell density in a perfusion process. Furthermore, a fed-batch feed strategy was introduced to keep the substrate concentration at a certain level. Cell density and viability were determined using a CEDEX system (Innovatis-Roche AG). Glucose and lactate were measured with an YSI 2700 Biochemistry Analyzer (YSI Life Sciences). Amino acids were quantified using an in-house developed HPLC method.

## Results

The FOGALE i-Biomass 465 sensor was used to monitor the viable cell density of different human, CHO and hybridoma cell cultures online. A good correlation of the permittivity signal and the offline measured viable cell density for the growth phase was verified (R > 0.99), but pH-shifts and increased cell aggregation had a negative impact on the correlation. The linear factor to calculate the viable cell density from the online permittivity signal varied between 4.5·10^5^ cells/(pF/cm) and 12.0 cells/(pF/cm). A clear relation between cell type (CHO, human or hybridoma) and the linear factor could not be established from the available data.

Subsequently, the online biomass monitoring system was used to carry out a 1 L spin-filter perfusion process with constant viable cell density at a predefined setpoint. The application of a permittivity closed-loop controlled cell bleed resulted in a steady concentration of 10^7^ viable cells/mL during perfusion, at a dilution rate of 1.0 d^-1^. As soon as this threshold was reached, the cell bleed was automatically started and controlled based on the online signal of the i-Biomass 465 sensor.

**Figure 1:**
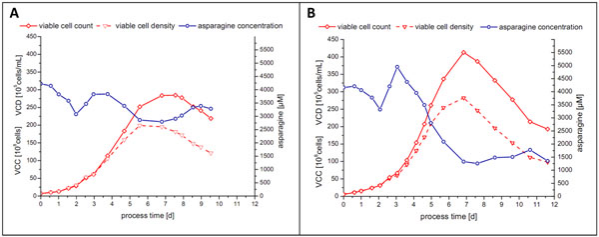
Total viable cell count, viable cell density and asparagine concentration of the two proof-of-concept CHO DP-12 fed-batch processes (initial working volume: 1 L; 37°C; 40% DO; pH 7.1).

In addition to the correlation with viable cell density, a linear relationship (R^2^ > 0.96) between the online i-Biomass 465 signal and the concentrations of numerous components, e.g. glucose, lactate, asparagine, glutamine, tyrosine, threonine, methionine, lysine, phenylalanine, serine, leucine and isoleucine, was found during the exponential growth phase of CHO-K1 and CHO DP-12 cultivations. The results indicated that the number of correlating substrates depended on the used cell line (CHO, human or hybridoma) and the process strategy (constant pH or pH-shift). Since, the established substrate correlations were more robust against process variations, they were investigated as a basis for a closed-loop feeding strategy in fed-batch cultivations. Compared to a pre-defined feeding schedule or to intermitted feeding this would have the advantage of avoiding nutrient limitations and substrate accumulation that might occur due to unexpected high or low cell growth. Also, feeding would be independent of human surveillance. The successful application of a completely automated permittivity-controlled feeding strategy was proved in two fed-batch runs with CHO DP-12 (ATCC CRL-12445) cells, as shown in Figure 1.

The feeding of both runs was controlled only through the permittivity signal in order to maintain the asparagine concentration at a certain level. Asparagine was chosen due to its central role in cell metabolism and to the fact that it is usually a limiting substrate in CHO DP-12 cultures. These proof-of-concept runs demonstrated that permittivity-based automated feeding can be a valuable tool for the optimization of fed-batch process parameters, such as feeding start, flow rate and composition.

## Conclusions

For suspension cultures with single cells and high viability a linear correlation (R^2^ ≥ 0.98) of the permittivity signal with the viable cell density measured offline was obtained, at least for the exponential growth phase. Based on this correlation, a closed-loop controlled cell bleed was implemented in a perfusion process in which the permittivity signal was used to keep the viable cell density at a constant level of 10^7^ viable cells/mL. Furthermore, a linear correlation with the i-Biomass 465 signal was observed for several substrates independent of the correlation between viable cell density and permittivity. Based on these results, a closed-loop controlled feeding was successfully established resulting in a fully automated fed-batch process.
